# Were ride-hailing fares affected by the COVID-19 pandemic? Empirical analyses in Atlanta and Boston

**DOI:** 10.1007/s11116-022-10349-x

**Published:** 2022-11-10

**Authors:** Tulio Silveira-Santos, Ana Belén Rodríguez González, Thais Rangel, Rubén Fernández Pozo, Jose Manuel Vassallo, Juan José Vinagre Díaz

**Affiliations:** 1 Transport Research Center (TRANSyT), Universidad Politécnica de Madrid, 28040 Madrid, Spain; 2 Group of Biometrics, Biosignals, Security and Smart Mobility (GB2S), Universidad Politécnica de Madrid, 28040 Madrid, Spain; 3 Department of Organizational Engineering, Business Administration and Statistics, Universidad Politécnica de Madrid, 28012 Madrid, Spain

**Keywords:** Ride-Hailing, Dynamic Pricing, Time Series Forecasting, Machine Learning, COVID-19, Transport Policy

## Abstract

Ride-hailing services such as Lyft, Uber, and Cabify operate through smartphone apps and are a popular and growing mobility option in cities around the world. These companies can adjust their fares in real time using dynamic algorithms to balance the needs of drivers and riders, but it is still scarcely known how prices evolve at any given time. This research analyzes ride-hailing fares before and during the COVID-19 pandemic, focusing on applications of time series forecasting and machine learning models that may be useful for transport policy purposes. The Lyft Application Programming Interface was used to collect data on Lyft ride supply in Atlanta and Boston over 2 years (2019 and 2020). The Facebook Prophet model was used for long-term prediction to analyze the trends and global evolution of Lyft fares, while the Random Forest model was used for short-term prediction of ride-hailing fares. The results indicate that ride-hailing fares are affected during the COVID-19 pandemic, with values in the year 2020 being lower than those predicted by the models. The effects of fare peaks, uncontrollable events, and the impact of COVID-19 cases are also investigated. This study comes up with crucial policy recommendations for the ride-hailing market to better understand, regulate and integrate these services.

## Introduction

Ride-hailing companies, which are also known as Transportation Network Companies (TNCs) in a broader sense, have become a common sight in cities around the world, and are one of the emerging mobility options that are revolutionizing door-to-door mobility services (Rangel et al. 2021). Ride-hailing companies such as Uber, Lyft, and Cabify use smartphone apps to provide their services, allowing the users to request a ride and receive information about the pick-up time, vehicle location, and the fare they will pay in advance (the app also makes payment easier for the users).

Ride-hailing companies are becoming increasingly popular because of their availability, convenience, and high quality of service (Rayle et al. 2016; Shokoohyar et al. 2020). These companies adjust their fares at any time using real-time dynamic algorithms (Chen and Sheldon 2015), whereas taxi fares are fixed and regulated. That means that when demand for ride-hailing services exceeds the supply of drivers, fares will automatically rise. Dynamic pricing (also known as *surge pricing*) is an automated system based on the basic principles of demand and supply. As a result, during times of high demand, passengers pay a higher fare for rides. When fares are increased dramatically due to high demand, users are generally notified before requesting a ride (Rangel et al. 2021).

Despite the growing popularity of ride-hailing companies, many of their operational metrics remain opaque. The public knows little about dynamic pricing and, as a result, understanding fares turn out troublesome. While it is unclear how these prices are updated at any given time, the ride-hailing market and its stakeholders can benefit from the overall short- and long-term fare prediction. Fares accurately predicted, can be used by: (i) TNCs to better understand how fares change over time; (ii) customers to search for the cheapest fares; and (iii) drivers to monitor fare increases for higher prices (and hence potential revenue to collect).

The COVID-19 pandemic is prompting a new scenario with new mobility lifestyles, in which public health and social distancing have become critical challenges. In addition to mobility restrictions, some areas have restricted the use of public transport after it was identified as a vector for the spread of infection in densely populated areas (Buja et al. 2020; Tian et al. 2020). Fear of infection also discourages people from using public transport (Wang 2014) and ride-hailing services (Shamshiripour et al. 2020), leading to a greater share in the use of active modes of transport (Abdullah et al. 2020; Bucsky 2020; de Haas et al. 2020).

There are changes in travelers’ and drivers’ mode choice behavior as a result of unusual occurrences such as technological emergence, pandemic conditions, etc. (Khoury et al. 2019). According to global economic reports, the global ride-hailing market would increase at a rate of 55.6% from 2020 to 2021 after the COVID-19 pandemic. From USD 75.39 billion in 2020, it is predicted to reach USD 117.34 billion by 2021. However, compared to the pre-COVID-19 estimate, the projection for 2021 is expected to be 2% lower (Shaheen et al. 2015). To reduce the potential of viral infection, initiatives such as creating barriers between the driver and the passenger, equipping the car with sanitizers, and installing digital thermometers to detect passengers’ body temperature may resuscitate the ride-hailing business (Khoury et al. 2019; Morshed et al. 2021). In that scenario, this paper addresses the following research question: “Were ride-hailing fares affected by the COVID-19 pandemic?”.

Thus, the objective of this paper is to investigate the impact of the COVID-19 pandemic on ride-hailing fares. To that end, the predictive capabilities of two models are analyzed: (i) a long-term prediction model (using a time series forecasting model); and (ii) a short-term prediction model (using a machine learning model). The difference between predicted 2020 fares trained on pre-COVID-19 data and actual 2020 fares can be used to analyze the pandemic’s impact. If indeed the COVID-19 pandemic affects ride-hailing fares, a long-term forecasting model trained on pre-pandemic data is likely to have difficulties when tested during the COVID-19 pandemic. Ride-hailing data was collected from the Lyft Application Programming Interface (API), collecting supply-side data in two urban areas in the United States (i.e. Atlanta and Boston) over 2 years (from January 1st, 2019 to December 31st, 2020).

On a general basis, this paper intends to: (i) propose a research design incorporating time series forecasting and machine learning models into the decision-making processes of agencies, stakeholders, and policymakers for the ride-hailing market; and (ii) explore pricing strategies on transportation systems and services (i.e., ride-hailing companies) through the use of novel models, for transport policy purposes.

To better understand the behavior of Lyft fares in the two cities, two different techniques (a time series forecasting model and a machine learning model) were employed. On the one hand, the Facebook Prophet model (a time series forecasting model) was used for long-term prediction to analyze trends and the global evolution of Lyft fares. On the other hand, the Random Forest model (a supervised machine learning model) was used for the short-term prediction of ride-hailing fares. Even though the two models have different approaches, the analysis of the fares predicted is complimentary. Furthermore, accurately forecasting ride-hailing fares is worth it for ride-hailing companies as they may provide information on demand peaks that these companies are currently unable to meet.

This paper presents some contributions to the ride-hailing market, as well as its stakeholders (e.g., drivers and customers). First, total fares were predicted, not just the surge multiplier (as noted by Battifarano and Qian 2019, who analyzed data before the COVID-19 pandemic). Second, two different short- and long-term prediction models were used to better understand the behavior of ride-hailing fares (focusing on applications of time series forecasting and machine learning models that may be useful for transport policy purposes). Third, data from two urban areas in the United States (namely Atlanta and Boston) collected over a long period (a total of two years) was used to calibrate the models. Fourth, the period analyzed includes the most critical phases of COVID-19, allowing for the detection of the pandemic’s impact on ride-hailing fares. Finally, it provides crucial policy recommendations for the ride-hailing market to better understand, regulate and integrate these services.

After the introduction, in the second section, the background and literature review are presented. The third section presents the two cities selected, followed by the fourth section describing the data for each case. In the fifth section, the methods used to obtain the results of this paper are discussed. In the sixth section, the results and discussion are presented, followed by conclusions and policy recommendations for the ride-hailing market.

## Background and literature review

The scientific literature on ride-hailing has grown in recent years in tandem with the global popularity of these services. The contribution to ride-hailing can be divided into two main categories: (i) studies focusing on demand (riders); and (ii) studies focusing on the supply side of ride-hailing (drivers), being demand the focus of most research studies.

Regarding demand, studies can be classified into two groups. The first set of contributions looks into ride-hailing users, both individually and in terms of trips, using data from surveys, as noted by Alemi et al. (2018) and Sikder (2019). According to Alemi et al. (2018), young people, people with a higher level of education, and people with a “technology-oriented” lifestyle are more likely to use on-demand ride services. Furthermore, residents of urban areas reporting a lower use of their cars compared to the past are more likely to adopt these services (Sikder 2019).

The second set of contributions uses empirical data to investigate the impact of ride-hailing services. Despite the limited amount of data available to date, several contributions are worth to be mentioned. To the best of the authors’ knowledge, at least three cities in the United States have publicly released ride-hailing trip data: Austin (Ride-Austin, 2017), Chicago (Chicago Data Portal, 2021), and New York City (TLC, 2020). Some studies have used these open-source databases to model the relationship between ride-hailing demand and other variables such as socioeconomic factors (Correa et al. 2017; Ghaffar et al. 2020; Yu and Peng 2019, 2020), built environmental factors (such as density, land use, infrastructure, and transit accessibility) (Gerte et al. 2018; Yu and Peng 2019, 2020), weather conditions (Ghaffar et al. 2020) and transit supply/service (Correa et al. 2017; Ghaffar et al. 2020; Lavieri et al. 2018; Soria et al. 2020; Yu and Peng 2020).

Aside from the above-mentioned studies, some researchers have focused their work on forecasting future demand. Time series models (Faghih et al. 2019) and machine learning models (Chen et al. 2021; Jin et al. 2020; Ke et al. 2017; Kontou et al. 2020; Yan et al. 2020) have both been used to predict ride-hailing demand. The operator can make real-time adjustments and assign drivers to riders based on the short-term prediction of ride-hailing demand, maximizing service and revenue.

Given the lack of data on ride-hailing supply, researchers have obtained primary data through APIs provided by operators. Several contributions have been made in various aspects of ride-hailing supply. Jiao (2018) and Hall et al. (2015), for example, studied dynamic pricing during a special event in Austin and New York City, respectively. In addition, Battifarano and Qian (2019) proposed a general framework for predicting the short-term evolution of surge multipliers in real-time, with their model predicting Uber surge multipliers in Pittsburgh up to two hours in advance.

Other aspects of the supply side include the analysis of ride-hailing fare patterns (Rangel et al. 2021), the impact of weather conditions on ride-hailing (Shokoohyar et al. 2020), the impact of ride-hailing systems on the traditional taxi sector (Akimova et al. 2020; Berger et al. 2018), and the comparison of the two services (Cramer and Krueger 2016; Rangel et al. 2021).

Given that COVID-19 is rapidly becoming a major global issue, it is worth mentioning a new set of contributions to the ride-hailing literature related to the pandemic’s drastic changes in people’s mobility habits. Data from travel behavior surveys has primarily been used to study the impact of the COVID-19 pandemic on the transport sector, particularly ride-hailing services. For example, there is evidence that the demand for public transport has decreased dramatically as a result of the higher risk of exposure when compared to other modes of transport (Bucsky 2020; Loa et al. 2021). Another study by Shamshiripour et al. (2020) investigated the perceived risk of different modes of transport in Chicago, finding that taxi and ride-hailing services are among the top three riskiest modes in people’s minds.

Individual modes of transport (such as private vehicles, cycling, and walking) appear to be more popular during the COVID-19 pandemic, whereas shared modes (such as public transport, ride-hailing, and taxi) appear to be less popular due to perceived risks. However, according to Loa et al. (2021), the COVID-19 pandemic has had only a short-term impact on ride-hailing frequency, and it is unclear whether the pandemic will have a long-term impact on ride-hailing usage in Toronto. Awad-Núñez et al. (2021) investigated people’s willingness to use and pay for public transport and shared mobility services (such as car-sharing, moped scooter-sharing, bike-sharing and kick scooter-sharing), as well as ride-hailing and taxi services in Spain. They concluded that in the post-COVID-19 phase, people’s willingness to pay for ride-hailing services is relatively high.

Another study was conducted by Du and Rakha (2020) during the COVID-19 pandemic to investigate ride-hailing trip changes depending on a range of variables. The Chicago Data Portal open database was used to collect empirical data for this study. The number of total trips, number of pooled trips, number of single trips, travel frequency, trip travel times, trip distances, and variations in longer trips across census tracts and shorter internal trips within a census tract were all investigated by these authors. According to the findings, the number of ride-hailing trips was significantly lower than those using personal vehicles during the COVID-19 pandemic. Due to less congestion, average travel distances became longer and average travel times shorter in most cases. In early March 2020, Uber and Lyft suspended trip pooling, thereby resulting in a significant drop in the number of pooled trips.

Despite the increasing interest in the impact of the COVID-19 pandemic on ride-hailing services, there are still some gaps in the literature. For example, no research has been done regarding the trends and changes in ride-hailing fares just before and during the COVID-19 pandemic. In addition, fare forecasting models should be investigated to improve long-term prediction (to analyze trends and global evolution of ride-hailing fares) and short-term prediction (to predict ride-hailing fares in a short period). These are the research gaps that this paper is attempting to tackle.

## Cities selected

This section provides a brief description of the cities selected to analyze the trends and evolution followed by ride-hailing fares before and during the COVID-19 pandemic. Atlanta and Boston were chosen since they are important employment centers in their respective regions with similar populations. However, they differ in urban morphology, transportation infrastructure, modal share, and other factors.

Atlanta, Georgia, has a population of 488,800 people (United States Census Bureau, 2019) and 5.3-million inhabitants in its metropolitan area (United States Census Bureau, 2010). Boston, Massachusetts, has a population of 684,379 people (United States Census Bureau, 2019) and 4.6 million inhabitants in its metropolitan area (United States Census Bureau, 2010). The city of Atlanta is nearly three times the surface size of the city of Boston with a lower population density (about 29% less) and a lower median household income (about 16% less). These cities, however, reveal some similarities, particularly in terms of poverty, bachelor’s degree or higher, and employment rates (United States Census Bureau, 2019).

Regarding mobility, the two cities are among the most congested ones in the world (Global Traffic Scorecard, 2020). The commute trips by mode for the cities of Atlanta and Boston are presented in Table 1. Only commuting mode shares are considered.

**Table 1:** Travel to work by city area 2014–2018. (Adapted from The Transport Politic, 2018)

Mode	Atlanta	Boston
Drive Alone (Personal vehicle)	77%	65%
Carpool	9%	7%
Transit	3%	14%
Walk	0%	1%
Bike	1%	6%
Other (e.g., Taxi, Lyft, Uber)	2%	2%
Work from Home	8%	5%

Even though non-drive-alone modes are not so representative in both cities, Boston has higher percentages of transit and active modes of transport (e.g., walking and cycling) compared to Atlanta. The use of ride-hailing services (such as Lyft and Uber) is similar in both cities, at around 2% or less.

Ride-hailing services are a popular and rapidly expanding mode of transport in cities all over the world. The two largest ride-hailing companies in the United States are Lyft and Uber. Nevertheless, this paper only focuses on Lyft services due to the lack of data available from other ride-hailing companies operating in the cities (see more details in Sect. 4 about data description).

Knowing how Lyft rides are calculated is important to better understand the cost of the ride. The Lyft ride price is comprised of the Lyft fare, tolls or local fees, and tips to the driver. Factors taken into account when estimating a fare are: (i) ride route; (ii) ride type; (iii) ride availability; and (iv) demand (Lyft, 2022). For Lyft, the service fee $p$ (total fare for a ride) is made up of two main parts (see Eq. 1).

1 $${p}_{service \, fee}= {p}_{base \, cost}+ {p}_{surge \, pricing}$$

The first component (base cost) includes regular fees such as one-off fees, service fees, and trip fees proportional to the trip’s duration and distance. The second component (surge pricing) reflects the result of Lyft’s surge pricing algorithm depending on supply and demand (S&D) imbalances (Schröder et al. 2020).

Lyft in the two cities offers a variety of service models (e.g., Lyft-type, Lyft Plus, and Lyft Lux), however, this paper primarily focuses on Lyft-type rides because it is the most popular service, which provides rides in regular vehicles for up to four people. Table 2 describes the factors that influence Lyft fares in Atlanta and Boston in 2021, based on regular service. The price of a Lyft ride varies across cities, as well as each aspect of the fee structure. Lyft fares are generally higher in large, high-density cities, with higher base fares, as is the case in Boston, for example.

**Table 2:** Factors that influence Lyft fares in Atlanta and Boston, based on regular service

Service fee	Variable	Atlanta	Boston
Base cost	One-off fee	USD 1.12	USD 2.18
	Service fee	USD 3.90	USD 2.35
	Cost per minute	USD 0.17	USD 0.37
	Cost per mile	USD 0.87	USD 0.92
Surge pricing*		S&D	S&D
Minimum fare		USD 5.30	USD 5.00

*Reflects the time evolution of supply and demand (S&D) imbalances

In the two cities, Lyft fares differ and are determined by the company’s policy, and the base cost considers the following factors: (i) the one-off fee, which remains constant regardless of the length or duration of the ride; (ii) the service fee, which is an additional fee added on a per-ride basis to support the Lyft Platform and related services (including a broad spectrum of operating costs and safety measures like insurance and background checks); (iii) the cost per minute; and (iii) the cost per mile. Surge pricing, also known as dynamic pricing, is a real-time dynamic algorithm used by their platforms to adjust prices (Chen and Sheldon 2015; Ngo 2015). Furthermore, the minimum fare is also included, which is a minimum fare for each service to compensate drivers in cases where short trips occur.

It is worth mentioning that the real-time dynamic algorithm (surge pricing) is not open data supplied by Lyft. This is the main reason why this research aims to better understand the behavior of ride-hailing fares, and how they are affected by the COVID-19 pandemic.

**Fig. 1:**
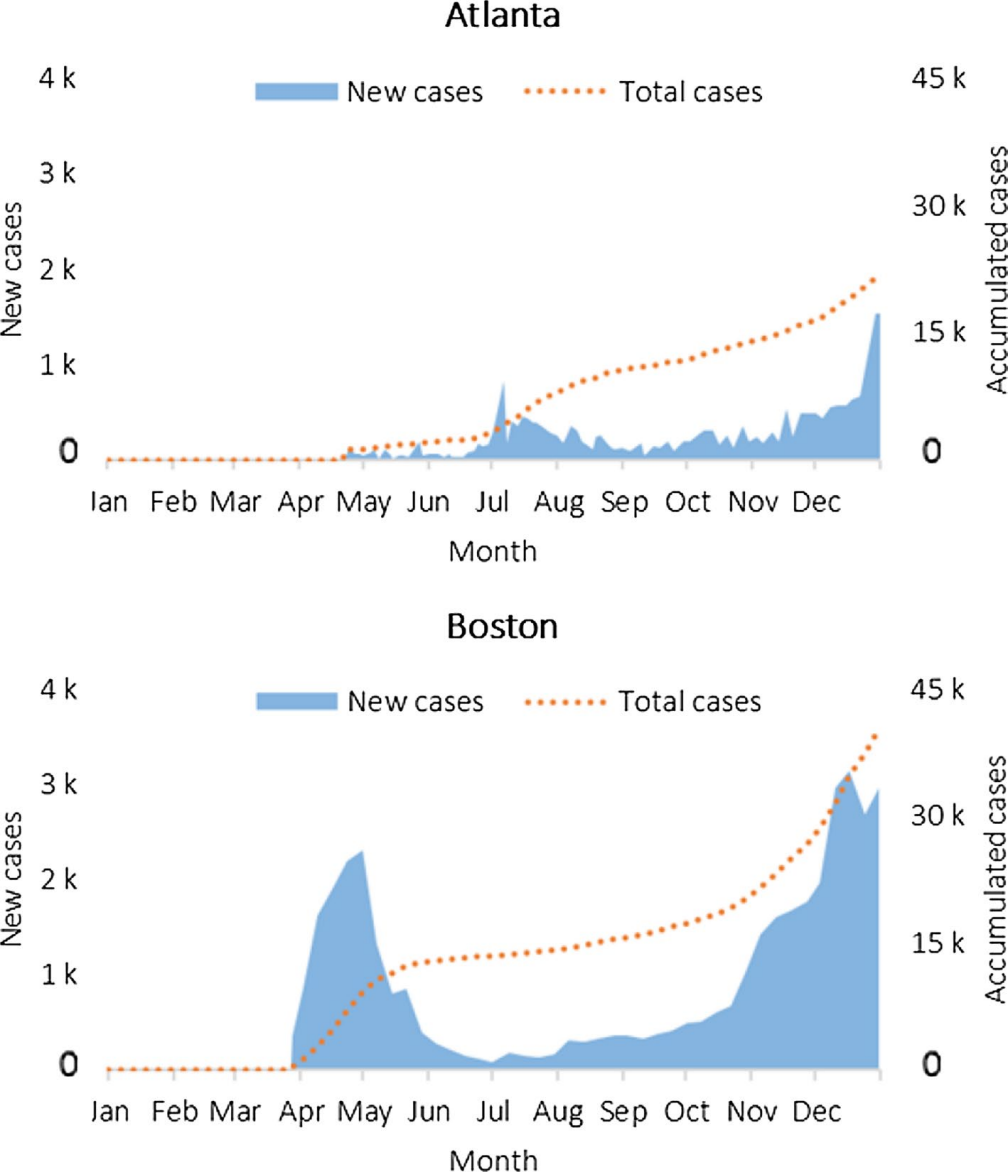
Evolution of COVID-19 cases during 2020 (Authors’ work based on the Fulton County Board of Health Epidemiology Division, 2022, and the Boston Public Health Commission, 2022)

In the United States, the first case of COVID-19 was confirmed on January 21st, 2020, and the World Health Organization (WHO) declared COVID-19 a “pandemic” on March 11st, 2020 (Javadinasr et al. 2022). The lockdown in Atlanta started on March 23rd, 2020 (City of Atlanta, 2022), while it started in Boston on March 16th, 2020 (City of Boston, 2021). Figure 1 shows the evolution of COVID-19 cases in these cities during 2020.

Atlanta reported a total of 21,486 COVID-19 cases in the year 2020, while Boston reported a total of 40,325 cases (almost twice as many as Atlanta). Atlanta’s peaks lag behind Boston’s, mostly occurring in July and December 2020, while Boston’s peaks have indeed occurred in May and December 2020.

Table 3 shows the impact of the pandemic on transport trends such as miles driven, travel times, and collisions in Atlanta and Boston, which are among the most congested cities in the world. Because of the COVID-19 pandemic, many people stopped sharing rides and avoided taking public transport. Overall, traffic congestion trends became “positive” as people largely avoided traffic jams associated with morning and afternoon commutes (Global Traffic Scorecard, 2020).

**Table 3:** Congestion rates in Atlanta and Boston. (Adapted from the Global Traffic Scorecard, 2020)

Congestion rates	Atlanta		Boston	
	2019	2020	2019	2020
Most congested city in the country	10th	22nd	1st	4th
Most congested city in the world	47th	174th	9th	36th
Hours lost in congestion	82 Hrs	20 Hrs	149 Hrs	48 Hrs
Cost of congestion per driver	USD 1214	USD 299.97	USD 2205	USD 710.57
Inner-city last-mile speed	12 MPH	19 MPH	12 MPH	15 MPH
Peak speeds	27 MPH	41 MPH	17 MPH	27 MPH
Off-peak speeds	45 MPH	48 MPH	36 MPH	37 MPH
Change in miles driven	–	−13%	–	−26%
Change in collisions	–	−25%	–	−33%

The city of Boston ranked first in 2019 as the most congested city in the United States, but saw 101 h saved in 2020, while the city of Atlanta saw only 62 h saved in 2020. Although Atlanta is less congested than Boston, the impact of COVID-19 on transport trends was relatively small in miles driven and collisions, which may be related to the lower supply of alternatives to driving, such as cycling and transit.

## Data description

This section presents the data used to explore the trends and evolution followed by ride-hailing fares before and during the COVID-19 pandemic. Data was collected using Lyft’s API in the two selected cities, Atlanta and Boston. It was not possible to obtain information from other ride-hailing companies operating in the cities since their APIs did not provide that information.

Using the web-scraping technique, a script was created in which the computer was taught to find the data that was deemed appropriate (Glez-Peña et al. 2013). These tools allow for the real-time collection of requested ride information while controlling for the latitude and longitude coordinates of the chosen origin and destination (OD) points.

**Fig. 2:**
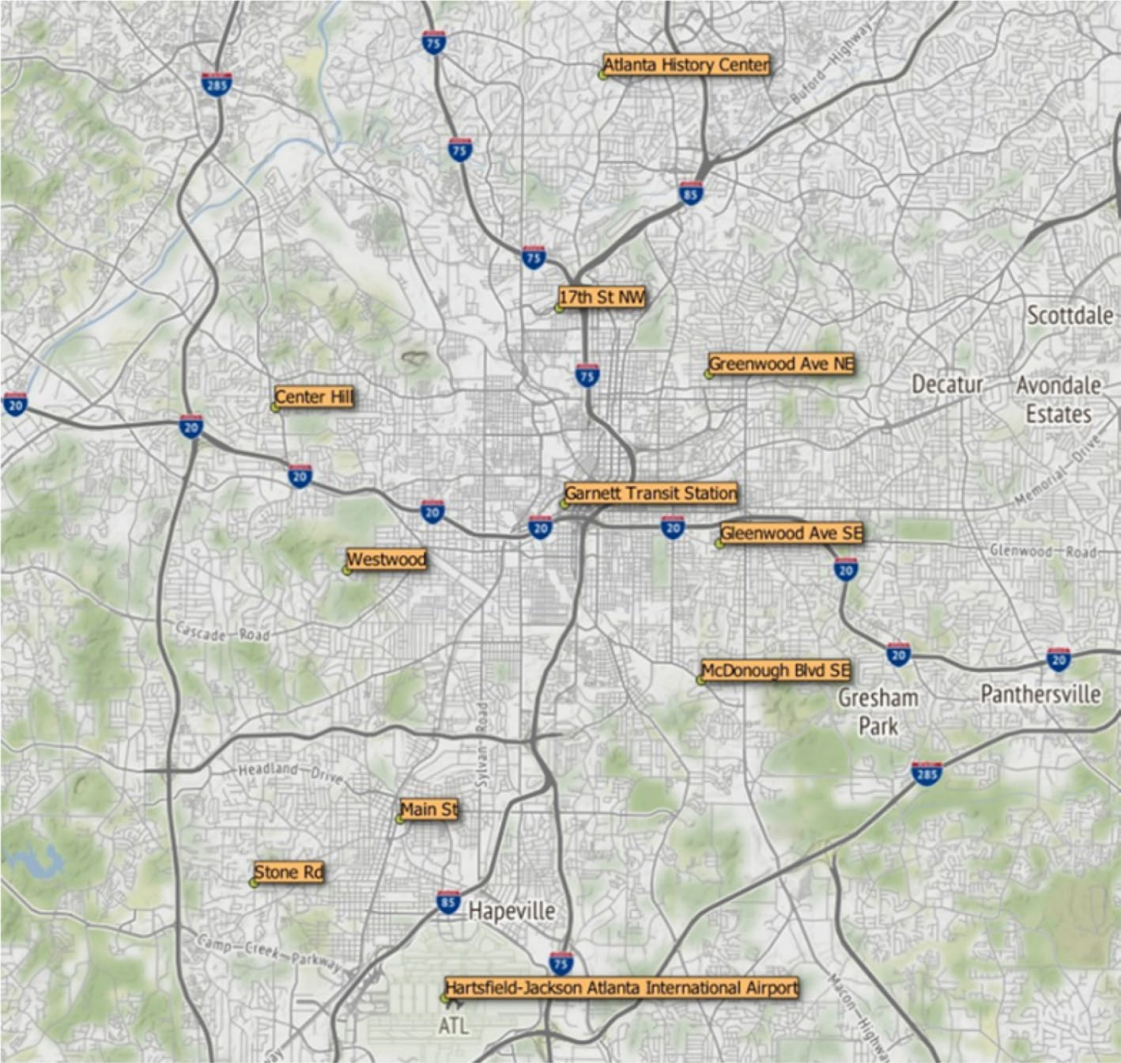
Atlanta city and selection of the ODs of the requested rides (Authors’ work using a GIS tool)

**Fig. 3:**
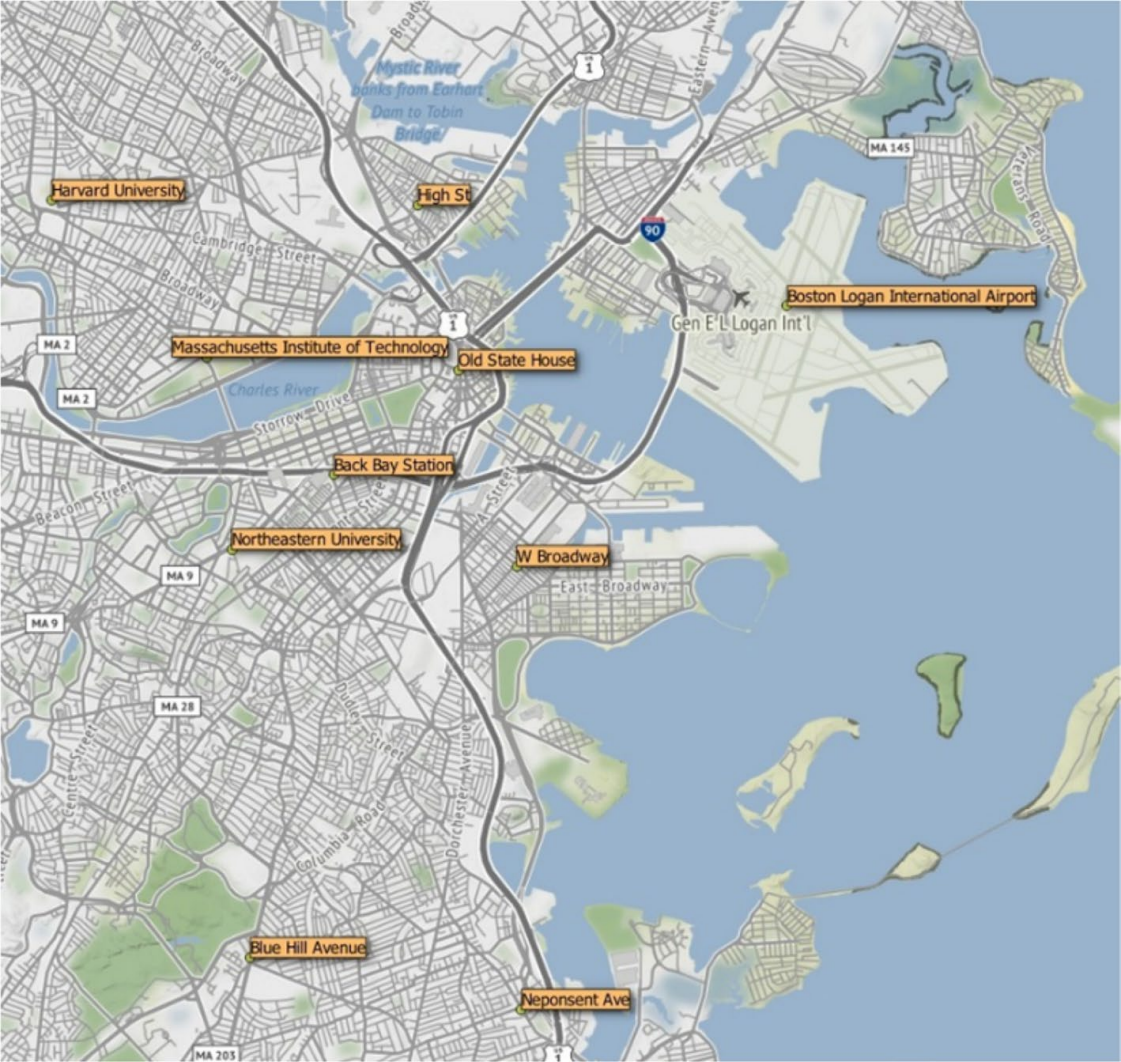
Boston city and selection of the ODs of the requested rides (Authors’ work using a GIS tool)

The ODs of the requested rides were defined at 11 locations in Atlanta (see Figs. 2) and 10 locations in Boston (see Fig. 3), which were used to collect information about ride-hailing fares. These locations were chosen to cover the two cities uniformly. The spots included high-demand locations (e.g., airports, public transport stations, tourist areas, etc.). In addition, various points in central and peripheral locations were chosen to provide a variety of routes (within the city center, from the periphery to the center, and from the periphery to the periphery).

Ride-hailing demand is high at two special locations in Atlanta (i.e., Hartsfield-Jackson Atlanta International Airport and Garnett Transit Station) and Boston (i.e., Boston Logan International Airport and Back Bay Station). Then, using a GIS tool, another nine points in Atlanta and eight points in Boston were chosen to uniformly cover the two cities, as previously stated. In the case of Boston, two points were chosen at Harvard University and Massachusetts Institute of Technology (MIT) university campuses, both of which are large academic centers with high demand located in the neighboring city of Cambridge. In the end, 11 locations in Atlanta were defined (making up a network with 110 potential routes) and 10 locations in Boston (defining a network with 90 potential routes).

For each ride requested through the Lyft API, the following data was gathered: (i) fare; (ii) trip distance; (iii) trip duration; and (iv) trip request time (with year, month, day, and hour information). The Lyft fare represents the cost of the ride as displayed by the app. The trip distance and duration indicate the distance and travel time required to travel to a specific OD, respectively. Note that the Lyft API’s estimated travel time is based on current traffic conditions.

Data were collected before and during the COVID-19 pandemic and stored at 1-hour intervals over 2 years (from January 1st, 2019 to December 31st, 2020), and 3,493,624 entries were collected from 3,508,800 potential inputs (731 days × 24 h × 200 routes in both cities). Then, a data processing process began, which included, for example, the verification of missing values and data cleaning. Data cleaning was required in cases where the fare, distance, and travel time variables had zero values. After the data cleaning process, the final dataset ended up containing 3,493,508 entries (with 99.56% of representativeness).

In both cities, the dataset’s representativeness is high, and the variation in the dataset’s size after the data cleaning process is negligible. A preliminary exploratory analysis of the sample was conducted after gathering all the necessary data for the study. Table 4 shows the descriptive statistics for the final data sample.

**Table 4:** Summary statistics of explanatory variables

Variable	Typology	Unit*	Summary statistics	Atlanta		Boston	
				2019	2020	2019	2020
Lyft fare (FARE)	Continuous	USD	Mean	16.973	19.851	18.713	19.010
			SD	5.577	7.793	8.139	7.939
			Min.	8.000	8.000	6.000	8.000
			25%	12.000	15.000	12.000	12.000
			50%	15.000	18.000	18.000	18.000
			75%	21.000	24.000	21.000	24.000
			Max.	140.000	142.000	210.000	160.000
Trip distance (DIST)	Continuous	Km	Mean	15.117	14.943	8.163	8.359
			SD	6.941	6.842	4.138	4.194
			Min.	3.975	4.506	1.819	1.867
			25%	9.479	9.447	4.731	4.876
			50%	14.307	14.033	7.322	7.500
			75%	19.425	19.119	11.024	11.121
			Max.	50.936	39.590	21.259	20.149
Travel time (TTIME)	Continuous	Minutes	Mean	20.094	18.292	18.371	17.914
			SD	7.360	6.131	8.494	9.204
			Min.	6.333	5.667	3.800	3.467
			25%	14.983	13.983	12.600	11.433
			50%	18.867	17.417	16.633	15.383
			75%	24.217	20.083	21.967	21.467
			Max.	73.250	71.867	103.817	86.067

* Although miles are commonly used in the USA, trip distance data has been converted to kilometers

As expected, the results show that the trip distance variable (DIST) is highly correlated with Lyft fare (FARE), being also the most significant variable for predicting ride-hailing fares. 

The year 2020 will be remembered for a global pandemic that devastated industries, businesses, and consumers, causing unprecedented economic and social disruption and reshaping people and goods movement across all modes of transport (Global Traffic Scorecard, 2020). As a result, many people stopped using ride-hailing services. Descriptive statistics show that in 2020 travel time duration decreased, implying a reduction of hours lost in traffic congestion, which is consistent with the literature (see Table 3). In addition, Lyft fares increased in the two cities. Although it is well known that Lyft uses dynamic pricing algorithms based primarily on the balance between supply and demand, along with competition with other services, empirical evidence on the main factors explaining fares is still limited.

**Fig. 4:**
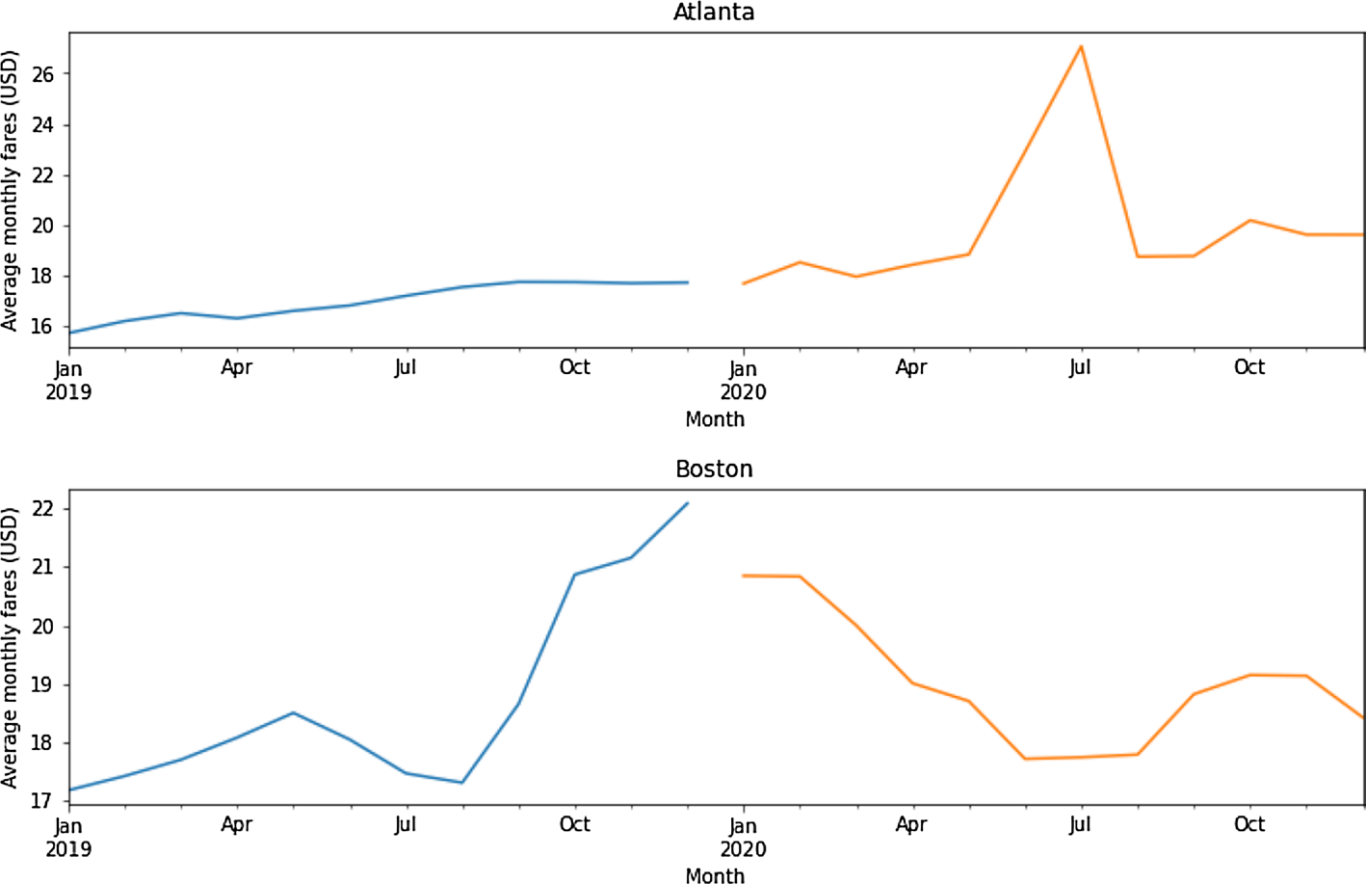
Average monthly fares for Lyft, before the COVID-19 pandemic (blue line) and during the COVID-19 pandemic (orange line)

Figure 4 presents the general trends of ride-hailing fares before and during the COVID-19 pandemic, with average monthly fares for each city.

**Fig. 5:**
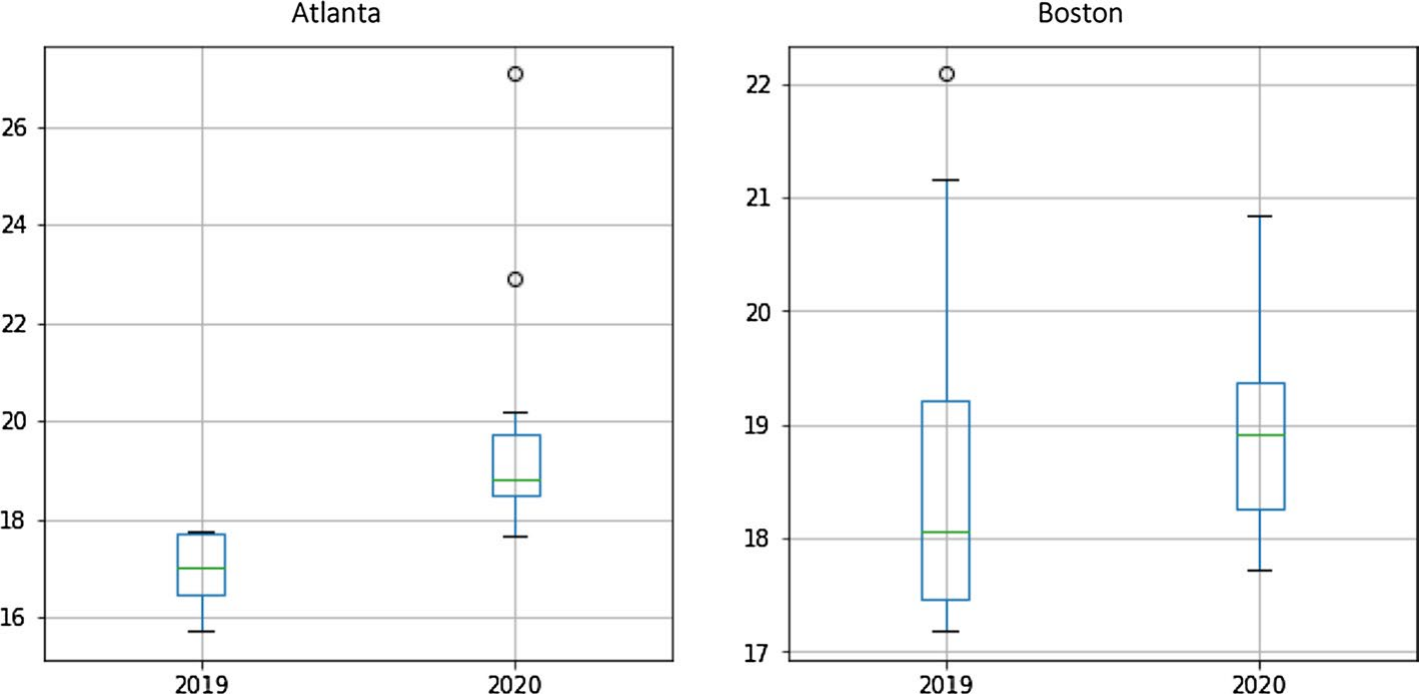
Boxplot of the average monthly fares for Lyft

Lyft’s fares rise in the year of the pandemic in the two cities, but with different trends. As fares in Atlanta peaked in June and July 2020 (see subsection 6.3 for a variety of potential causes), fares in Boston showed a downward trend during this period. Figure 5 shows the boxplot graph for each city over the two years using the average monthly fares for Lyft in Atlanta and Boston. It should be noted that during the data cleaning process, no outliers of the Lyft fares were removed.

## Methods

This section describes the methods used to explore the trends and evolution followed by ride-hailing fares before and during the COVID-19 pandemic, through a time series forecasting model (for long-term prediction) and the use of a machine learning model (for short-term prediction).

### Time series forecasting for long-term prediction

Predicting fares is key for business management. The time series model is used for forecasting purposes where time is a significant factor. It is also important because many predictions involve time-related components that must be carefully handled when the actual outcome is undetermined. Knowing the pattern of related data and their time is required to identify the root cause of a specific event (Kumar Jha and Pande 2021).

The following are the four major components of time series: (i) level, which is the baseline for time-series data; (ii) trend, which is represented by a curve that may increase or decrease over time; (iii) seasonality, which is represented by a cycle or pattern over time; and (iv) noise, which represents variation in the observed data. The interactions between these components are typically classified as an additive (see Eq. 2) or multiplicative (see Eq. 3) model

2 $$y\left(t\right)=Level+ Trend+Seasonality + Noise$$.

3 $$y\left(t\right)=Level \times\, Trend \times\, Seasonality \times \, Noise$$

On the one hand, in an additive time series, the components add up to form the time series, and the amplitude of seasonality is maintained as the trend increases. On the other hand, in a multiplicative time series, the components multiply to form the time series, and the amplitude of seasonality also increases with the trend. This research suggests testing additive and multiplicative models in each selected city, using the one with the best performance metric.

 Because forecasting is frequently the primary goal of time series analysis, predictive accuracy must be evaluated. In most cases, accuracy measures how well the model reproduces traditionally collected data (goodness-of-fit). The forecasting error (difference between the actual and predicted values) is frequently used as a measure of accuracy. The Root Mean Squared Error (RMSE) and the Mean Absolute Percentage Error (MAPE) are two commonly used metrics for evaluating the accuracy of forecasting models (Washington et al. 2011).

 The Root Mean Square Error (RMSE) metric is an error validation metric that measures the difference between real and predicted values. The difference is known as residuals, which are calculated from the standard deviation of the prediction errors (see Eq. 4). This metric uses the same dependent unit. In this paper, the dependent unit is the United States currency unit (USD), which corresponds to the Lyft fare validation error.

4 $$RMSE = \sqrt{{\sum }_{i=1}^{N}\frac{{({Actual}_{i}- {Predicted}_{i})}^{2}}{N}}$$

Second, the Mean Absolute Percentage Error (MAPE) is a statistic that measures the accuracy of a forecasting method. The MAPE is usually expressed as a percentage (see Eq. 5).

5 $$MAPE = \frac{1}{N}{\sum }_{i=1}^{N}\left|\frac{{Actual}_{i}- {Predicted}_{i}}{{Actual}_{i}}\right|$$

In 2017, Facebook launched Facebook Prophet, an open-source forecasting tool for making business predictions, which is used in this paper to estimate ride-hailing fares in the long term. This is a novel model for forecasting time series data that fits nonlinear trends with annual, weekly, and daily seasonality, as well as holiday effects. It works best with time series with strong seasonal effects and historical data from multiple seasons. Prophet is resistant to missing data and trend shifts, and usually handles outliers well (Chikkakrishna et al. 2019; Kumar Jha and Pande 2021).

According to Yang et al. (2019), the following are the most important advantages of using the Facebook Prophet model:

- The model can be easily adjusted to take into account a variety of seasonal and trend changes.
- The fitting is very fast. Given that hundreds of models will be trained; this is a critical feature.
- Since each parameter’s role is clear, tuning it results in understandable changes in the solution.
- When regular sampling overtime is not required, sparse missing values do not need to be interpolated.

The Facebook Prophet model has proved to be useful in predicting travel behavior in recent years, especially in the field of transport. For example, Chikkakrishna et al. (2019) present a short-term traffic prediction study using Facebook Prophet, which was used to estimate traffic volumes, as well as allow unsmoothed data to better fit models. Another example is the study by Pontoh et al. (2021), which used Facebook Prophet to predict the monthly number of train passengers and to automatically detect changes in trends and seasonal patterns.

The Facebook Prophet model was chosen in this paper to predict ride-hailing fares in Atlanta and Boston over a long period because it is an emerging technique with broad applicability. The entire approach for applying this model is presented in the results section (see subsection 6.1).

### Machine learning model for short-term prediction

Machine learning techniques offer advantages for extracting information from data and representing complex relationships in a data-driven manner. The Random Forest model, a machine learning technique, was used in this paper to predict the ride-hailing fare in the short term. Random Forest is a supervised learning algorithm that uses an ensemble of tree-structured learners (i.e., decision trees) to combine their predictions and generate a final prediction (Breiman 2001). Each base learner is a regression tree to predict a continuous outcome variable for regression problems (i.e., the response variable is continuous rather than categorical).

According to Yan et al. (2020), the following are the most important advantages of using the Random Forest model:

- It is one of the most accurate general-purpose machine learning methods because of its ability to model complex nonlinear relationships between the input variables and the response variable.
- It is sufficiently robust since its input variables can be of any type (numerical, categorical, continuous, or discrete), and it is unaffected by skewed distributions, outliers, missing values, or irrelevant variables.
- It can limit overfitting without significantly increasing error due to bias.
- It requires only minor hyper-parameter tuning and is usually unaffected by their values to achieve good performance.
- It requires a short amount of training time.

The Random Forest model’s usefulness in predicting travel behavior has been demonstrated in recent years, particularly in the field of transport. Cheng et al. (2019) present a review of recent studies that use the Random Forest method to solve transport forecasting and classification problems, which are divided into four categories: (i) travel choice behavior; (ii) traffic incident predicting; (iii) traffic time/flow prediction; and (iv) pattern recognition.

Yan et al. (2020), for example, used the Random Forest to model ride-sourcing demand in Chicago, comparing predictive capabilities to those of the classic multiplicative model, finding that Random Forest is superior in terms of predictive accuracy and model fit (which can be calculated using Eq. 4 and/or Eq. 5). These results demonstrate how machine learning techniques can be used to improve travel demand forecasting. The Random Forest method also considers the importance of the input variables.

In this paper, the Random Forest was used to predict fares before and during the COVID-19 pandemic, as well as to identify the main issues to reach a greater prediction accuracy. This model was chosen to predict ride-hailing fares for the Lyft service in cities selected (one-hour forecast horizon) due to its strengths and wide applicability. Several input features were used (see data described in Sect. 4), including time data (with month, day, and hour information) and ride supply information (fare, trip distance, and trip duration) in the previous three hours to train two models: (i) one model for 2019, before the outbreak of COVID-19; and (ii) a model for 2020, during the pandemic.

To account for the seasonal effect, the model was trained using data from the first 21 days of each month of the year, with the remaining days of each month being used for testing. All experiments were subjected to a 5-fold cross-validation, with four of the five partitions being used to train the classifier and the remaining used to test the results. In addition, the random partition process, which included five independent runs of 5-fold cross-validation, eliminated potential biasing and overfitting effects. The following configuration was chosen for training the Random Forest models after a hyperparameter tuning process:

- Number of trees in the forest (*n_estimators*): 30.
- The maximum number of splits of the tree (*max_num_splits*): 10.
- The minimum number of samples to split an internal node (*min_samples_split*): 2.
- The minimum number of samples for each leaf node (*min_samples_leaf*): 4.
- Number of features considered for splitting a node (*max_features*): 12.

## Results and discussion

This section summarizes the main findings of the analyses conducted in this research. The results from the two techniques are presented, involving the application of the Facebook Prophet model and the Random Forest model, followed by the discussion.

### Application of the Facebook Prophet model for long-term prediction

The Facebook Prophet model was applied to predict Lyft fares for the COVID-19 pandemic period (which began in mid-March 2020 in the two cities). For each city, the 2019 dataset (also known as the base year) was used for training and testing the model, and future Lyft fares were then estimated and compared to the year 2020 (also known as the comparison year) using model performance metrics. Each dataset (base year and comparison year) was converted into time series using two key features of the model: (i) the trip request time, which is a date-time type that assigns the data a certain sequence or order, and (ii) the Lyft fare, which is the target variable to be forecasted.

The data was grouped following an approach with spatial aggregation. Seasonal decomposition calculated using moving averages for Atlanta and Boston before and during the COVID-19 pandemic is presented in the Appendix. The seasonal decomposition returns the average hourly fare values of all routes for 2019 and 2020, as well as the objects with the seasonal trend and residual attributes. Average Lyft fares increased in the year of the pandemic in the two cities, but with different trends, as mentioned before. In the case of Atlanta, the evolution of Lyft fare in the 2020 pandemic year follows nearly a linear trend, with exceptional behavior in June and July 2020 (see subsection 6.3 for a variety of potential causes). However, during that year in Boston, there was a downward trend with common values.

The seasonality of the time series in each period was verified using a statistical test based on the Augmented Dickey-Fuller test (Mushtaq 2011), as shown in Table 5, with the following hypotheses: (i) Null Hypothesis – H_0_: Failure to reject the null hypothesis indicates that the series is not stationary, that is, it has a time-dependent structure; and (ii) Alternative Hypothesis – H_1_: The null hypothesis is rejected, implying that the time series is stationary, without any time-dependent structure.

**Table 5:** Statistical Test – Augmented Dickey-Fuller test

Statistical Test	Atlanta		Boston	
	2019	2020	2019	2020
ADF test	−3.998	−3.525	−5.709	−4.773
*p*-value	0.001	0.007	0.000	0.000

**Fig. 6:**
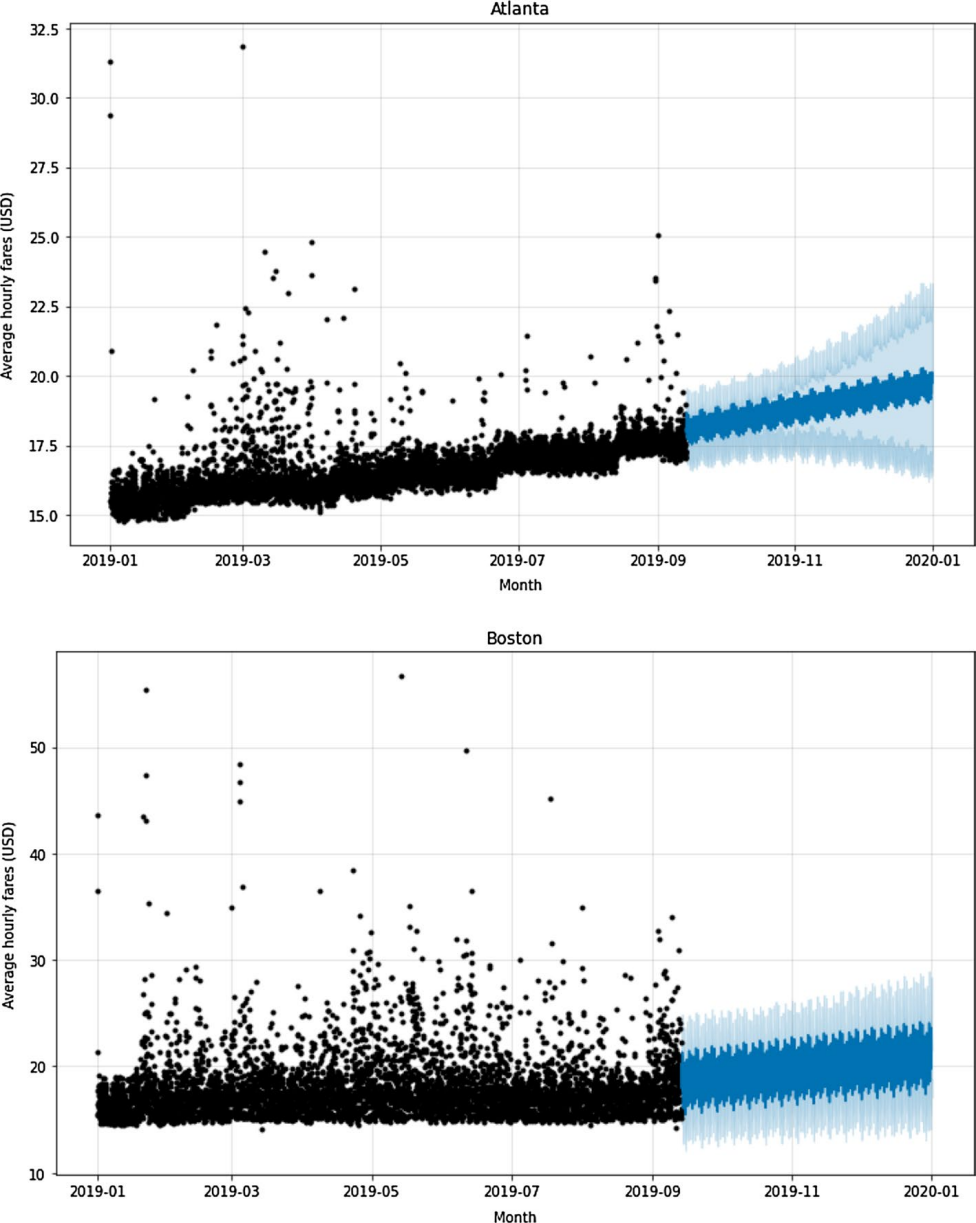
Training (in black) and testing (in blue) of the Facebook Prophet model in the base year (2019)

The p-values were all below 0.050, indicating that the null hypothesis (H_0_) was rejected, so all series are considered stationary. Then, the Facebook Prophet model was applied. The model was trained and tested with a train/test ratio of 70/30 using the 2019 dataset as the base year. The data training sample was from January to mid-September, and testing occurred between mid-September and December. The results of this model for the two cities are shown in Fig. 6.

In Atlanta and Boston, the results reveal a multiplicative model, in which the trend and seasonal components are multiplied and then added to the error component. In each city, both additive and multiplicative models were tested, to select the one with the best performance metric. Table 6 shows a comparison of the performance metrics of the models, in each city, using Train/Test in the base year (2019).

**Table 6:** Comparison of model performance metrics – Train/Test in the base year (2019)

Performance metrics	Atlanta	Boston
Root Mean Square Error (RMSE)	USD 1.370	USD 3.458
Mean Absolute Percentage Error (MAPE)	6.561%	8.829%

The models produced reasonable results in the RMSE and MAPE metrics. The performance is better in the case of Atlanta compared to Boston, which may be related to the fact that surges and outliers of the Lyft fare in 2019 are higher in Boston than in Atlanta (see Figs. 4 and 5)

**Fig. 7:**
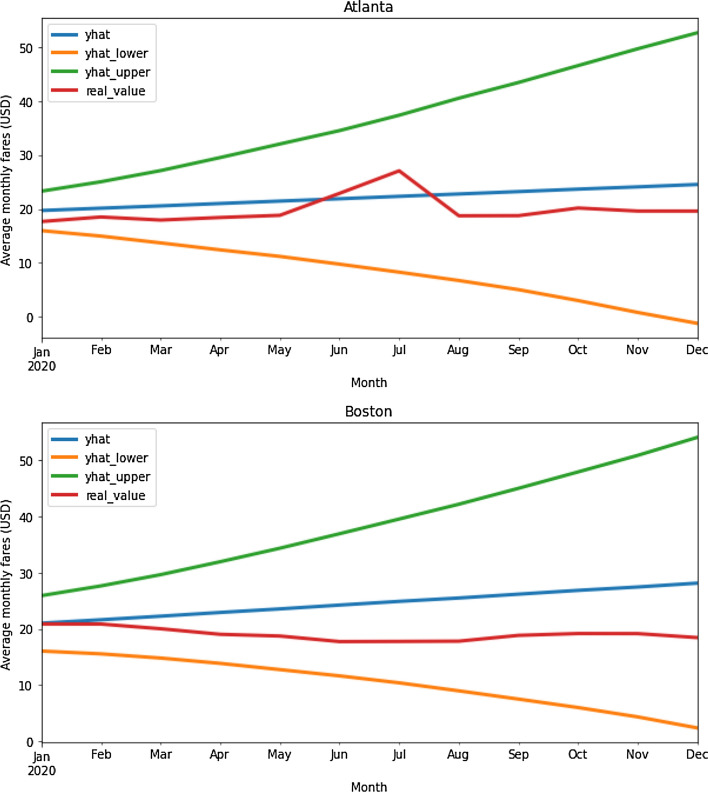
Estimated average monthly fares for Lyft in comparison to 2020

Lyft’s future fares for the comparison year (2020) were predicted using average hourly fares obtained from training and testing the Facebook Prophet model in Atlanta and Boston in the base year (2019), following what was shown in Fig. 6. However, to better understand the trends and evolution of Lyft’s fares, the results of the predicted values were grouped into average monthly fares for the year 2020 and compared with actual values for the same year (see Fig. 7).

The Facebook Prophet model presents the results of the Lyft fare forecast for the year 2020 (represented by the blue line), as well as the lower and upper bounds of the uncertainty interval around the final prediction (represented by the orange and green lines, respectively). The real values of Lyft’s fares in the year of the pandemic (represented by the red line) follow different trends in the two cities. The lockdown due to the COVID-19 pandemic began in mid-March in both cities (see Sect. 3), and the predicted results are similar to those observed in January and February 2020, particularly in Boston. In the following months (mid-March to December 2020), the values observed in the 2020 pandemic year are lower than the ones predicted, which positively proves the research question in this paper, that ride-hailing fares were affected by the COVID-19 pandemic. The predicted values are closer to the actual values for the city of Atlanta, which has half the total reported COVID-19 cases as Boston (see Fig. 1). However, it is difficult to predict uncontrolled situations, especially in Atlanta, as Lyft’s fares grow and reach a peak in June and July 2020), extrapolating the predicted values of the model (see subsection 6.3 for a variety of potential causes).

### Application of the Random Forest model for short-term prediction

This subsection shows how the Random Forest model was used to predict the expected fare for the Lyft service in Atlanta and Boston with a one-hour prediction horizon. Several input features were used (see data described in Sect. 4), including time data and ride supply information in the previous three hours to train two models: (i) one model for 2019, before the outbreak of the COVID-19 pandemic; and (ii) a model for 2020, during the COVID-19 pandemic.

The MAPE and RMSE metrics (see Eq. 4 and Eq. 5, respectively) were used to assess the model’s performance and compare it with other models in different scenarios. Table 7 shows the model forecast results, which group all potential routes from each city (spatial aggregation).

**Table 7:** Prediction results of the Random Forest approach for all routes in each city

City	Train/Test in 2019		Train/Test in 2020	
	MAPE (%)	RMSE (USD)	MAPE (%)	RMSE (USD)
Atlanta	1.570	0.622	3.970	2.052
Boston	5.920	2.077	1.530	0.760

The MAPE results show how the Random Forest model performs well, with low percentage errors (below 1.6%) for Atlanta in 2019 and Boston in 2020, and slightly worse results for Atlanta in 2020 (3.970%) and Boston in 2019 (5.920%). In the case of Atlanta, worse performance was achieved in 2020, which could be related to the difficulties in predicting the large fare increase in June and July 2020 (see Fig. 4). In the case of Boston in 2019, fare peaks (“surges”) were quite common, being difficult to predict by the model. It appears that the Random Forest model has problems predicting surges caused by supply and demand imbalances. That is the main reason for errors in the overall performance. According to Battifarano and Qian (2019), these changes can be extremely strong (up to eight times higher than the basic price) and rapid (less than an hour), making forecasting difficult and the model tending to underestimate fare peaks.

The Random Forest approach was also applied to each of the potential routes of the two cities to deepen the analysis. This was done with the understanding that short-term forecasting on specific routes can benefit both customers (who may have better knowledge to plan their trips) and drivers (who can increase their earnings by looking for routes with higher fares). A specific Random Forest model was retrained (in years 2019 and 2020) for each potential route in the two cities (i.e., 110 routes in Atlanta and 90 routes in Boston) using the same set of hyperparameters.

**Fig. 8:**
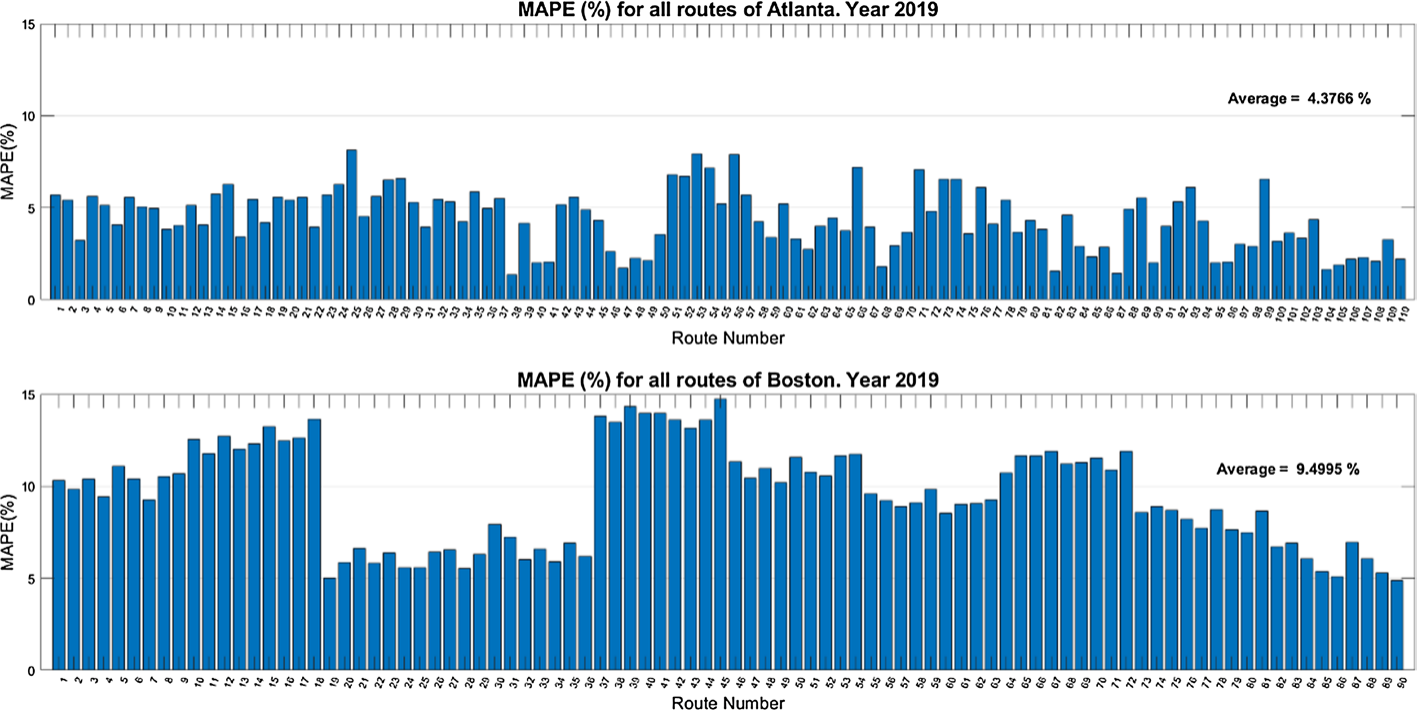
Predictions for each possible route for the year 2019

The MAPE metric was used to assess performance and predict results for each city using training and testing data from 2019 (before the outbreak of the COVID-19 pandemic). The results are shown in Fig. 8.

The 2019 prediction results in Atlanta and Boston for each possible route are reasonable, though they perform worse than in the case of the spatial aggregation modeling (see Table 7). The MAPE average values are better in Atlanta (4.38%) than in Boston (9.50%), which again could be due to the difficulty of predicting surges, which are much more common in Boston.

In the case of Boston, “groups” of consecutive routes with similar performance are also noticed. Routes 19 to 36, for example, have MAPE values of around 5%, noting that they are all close to the city center (the first nine departing from the High Street and the next nine departing from the Old State House). These routes also have the best prediction results because they are less likely to experience surges. However, in the case of Atlanta, there are no such groups of routes with similar performances because the frequency of these peaks is not as important and remains reasonably steady throughout all routes.

**Fig. 9:**
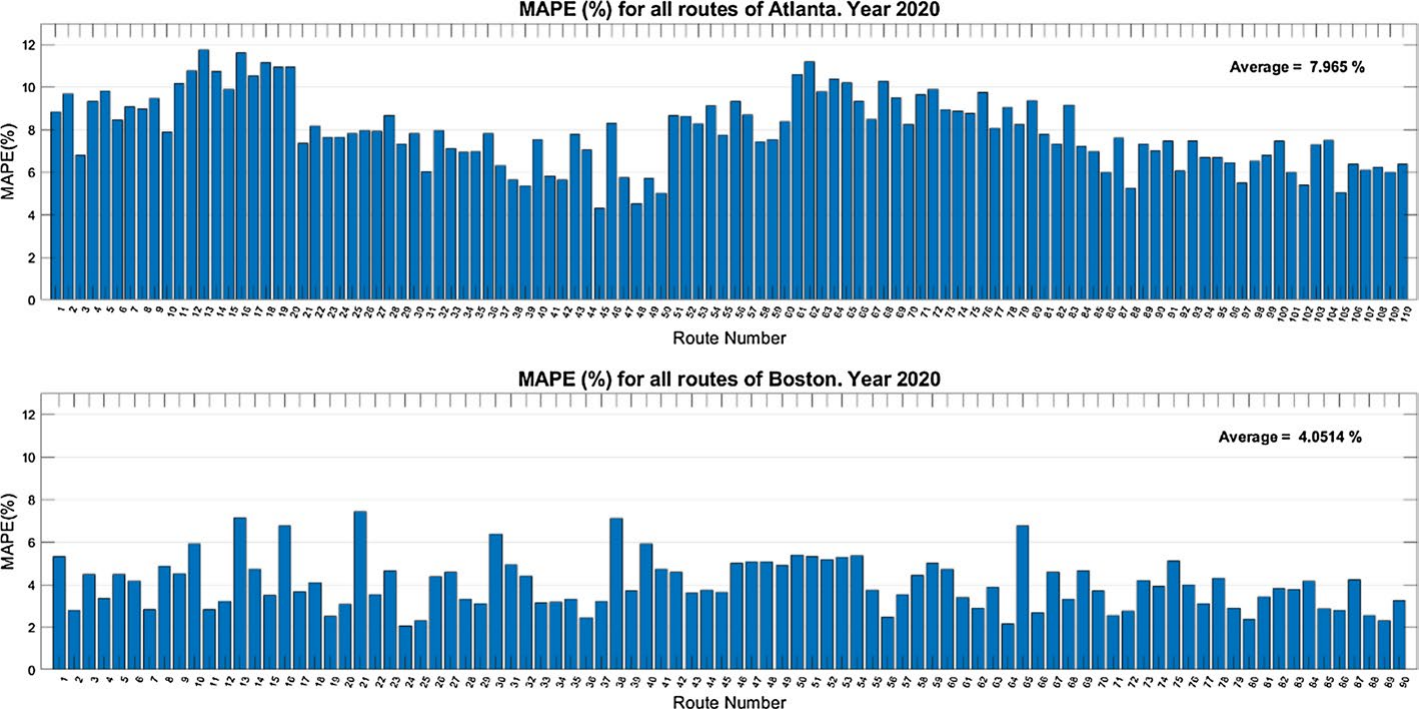
Predictions for each possible route for the year 2020

**Fig. 10:**
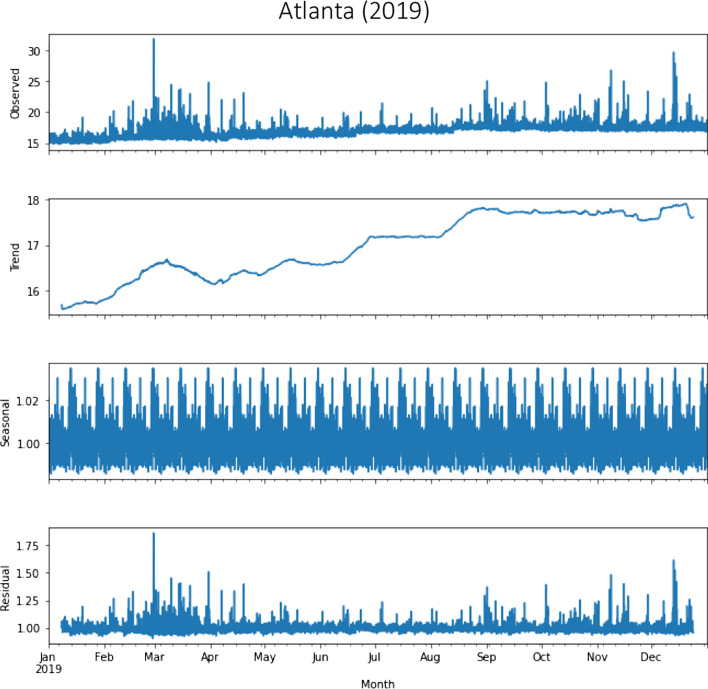
Seasonal decomposition using moving averages in Atlanta in the year 2019

Figure 9 shows the results of modeling per specific route in the two cities using training and testing data from the year 2020 (during the COVID-19 pandemic).

The COVID-19 pandemic marked the year 2020, but the models still have a relatively good performance (with MAPE metrics below 10%). In this case, the average MAPE value in Boston (4.05%) is slightly better than in Atlanta (7.96%). In the pandemic year, the surging-price factor is less important, and Atlanta’s worst performance is likely due to difficulties in predicting significant and long-term fare increases in June and July 2020 (see Fig. 4), with possible causes discussed in the next subsection. In light of these results, it seems clear the need of training specific Random Forest models for the pandemic situation to assure good performance in fares prediction.

### Discussion

The main findings of the prediction results of the models used in this paper are discussed in this subsection, particularly the possible causes of prediction problems (peaks in the fares curves) and/or uncontrolled situations. Using different approaches, two forecasting models (Facebook Prophet and Random Forest models) were used to verify trends and developments in ride-hailing fares before and during the COVID-19 pandemic. It is noteworthy that these models contribute to achieving a better understanding of the behavior of Lyft fares in Atlanta and Boston in a complementary way.

The Facebook Prophet model was applied for long-term prediction to analyze trends and the global evolution of Lyft fares. With this method, Lyft fares were predicted using a time series model with data before the pandemic, and the results were compared to current values observed over the pandemic period (see Fig. 7).

On the other hand, the Random Forest model (a supervised machine learning model) was applied for the short-term prediction of ride-hailing fares. This type of prediction can help vacant drivers move from over-supply to over-demand regions. To that end, several input features (including time data and ride supply information from the previous three hours) were used to train a model for 2019 (before the COVID-19 outbreak) and another model for 2020 (during the pandemic), as shown in Table 7.

In addition, both models used spatial aggregation, primarily for long-term prediction (since the goal was to obtain general fare trends), though the short-term prediction also included forecasts for specific routes (see Figs. 8 and 9). This can benefit both customers (who may have more knowledge to plan their trips) and drivers (who can increase their earnings by getting to know routes with higher fares).

The results of the two models show that during the COVID-19 pandemic, ride-hailing fares were highly affected, confirming the research question in this paper (namely, “Were ride-hailing fares affected by the COVID-19 pandemic?”). Furthermore, both models produced reasonable results according to the performance metrics used (see Tables 6 and 7), but it was difficult to predict the impact of the following cases: (i) fare peaks; (ii) uncontrollable events; and (iii) COVID-19 cases. The first case occurred primarily in Boston in 2019, due to the difficulty in predicting surges (see Fig. 8). The second case occurred mainly in Atlanta in 2020, with fares increasing and peaking in June and July 2020 (see Fig. 4 and Appendix), due to the occurrence of uncontrollable events. The third case occurs mainly in the city of Boston, which has almost doubled the total number of COVID-19 cases recorded in Atlanta in 2020 (see Fig. 1), and with fare predictions that are less close to the actual values (see Fig. 7).

Possible causes of these uncontrollable events include George Floyd’s racial protests in Georgia, United States (The New York Times, 2020), as well as the death of a black man at the hands of an Atlanta police officer (USA Today, 2020; WSB-TV, 2020). Protesters stopped traffic on several roads in Atlanta, including Interstate-75, which crosses from north to south and even passes through the city center (see Fig. 2) and is ranked 10th among the top 25 worst corridors in the country (Global Traffic Scorecard, 2020). According to the 2019 American Community Survey, the city of Atlanta has a 51.0% of black population, twice as much as Boston (United States Census Bureau, 2019).

## Conclusions and policy recommendations for the ride-hailing market

This research examines ride-hailing fares before and during the COVID-19 pandemic using explanatory variables such as fare, trip distance, trip duration, and trip request time (with year, month, day, and hour information). To that end, two techniques were used: The Facebook Prophet model for long-term prediction and the Random Forest model for short-term prediction. Both models were applied in two urban areas in the United States (namely, Atlanta and Boston).

Considering the lack of up-to-date official empirical data on ride-hailing demand, at least in the cities selected, ride-hailing fares can provide a reasonable proxy for estimating demand levels. The authors recommend that transport authorities should require ride-hailing companies operating in their regions to provide data on fares to better understand, regulate, and integrate these services with other transport modes, particularly public transportation.

The findings reveal that each model provides a complementary approach to understanding Lyft fares. The results of both models indicate that ride-hailing fares were affected during COVID-19, with values in 2020 being lower than those predicted using 2019 data. Although the two models have different approaches, both produced reasonable performance metrics (particularly the MAPE metric, with values below 10% in both cases). However, both models had problems predicting the impact in some circumstances (e.g., fare peaks, unexpected events, and the impact of COVID-19 cases). Difficulties to estimate fare peaks were particularly noticeable in Boston in the year 2019, due to the frequent sudden fare surges. A relevant unexpected event happened in Atlanta in 2020, with fares increasing and peaking in June and July of that year due to racial protests that prompted the closure of the main highways, disrupting ride-hailing services and fares. Ride-hailing fare predictions were also less close to the actual values in the city of Boston, which has nearly doubled the total number of COVID-19 cases reported in Atlanta in 2020.

From a transportation policy perspective, the authors highlight several benefits of knowing/predicting ride-hailing fares for different stakeholders, especially: (i) public authorities; (ii) regulatory agencies; (iii) TNCs; (iv) customers; and (v) drivers.

Knowing and predicting ride-hailing fares can help public authorities establish and implement policy measures to create a fair competitive framework with the taxi industry. It is also up to public authorities to find ways to promote greater coordination of ride-hailing services with other transport modes to promote their use where they can be most effective, incentivizing the connection with the public transportation network to achieve maximum global welfare. The techniques and findings of this paper can be used by regulatory agencies to ensure fair competition among TNCs. The findings may also assist them in identifying bad practices used among operators who seek to gain a dominant position to increase their earnings.

TNCs could apply the techniques and findings of this paper to better match supply and demand (e.g., the work schedule of drivers, and the location of vehicles closer to the places with more demand). The findings can assist customers in determining the cost of a ride in advance, allowing them to select the most cost-effective alternative for their trips based on their priorities. They can also help drivers keep track of fare increases to secure higher pricing and thus potentially larger earnings.

To sum up, better regulation in the ride-hailing sector is important for different stakeholders, not only in the United States but all around the world. This research covers a wide range of topics and provides numerous opportunities for future research, such as: (i) using these methods to compare the different phases of the pandemic as well as the post-pandemic scenario to track changes in ride-hailing fares; (ii) extending the research methods to other urban areas and/or geographies to gain a broader perspective and compare the results to the findings of this paper; and (iii) comparing the results of other artificial intelligence models (e.g., using other machine learning models) for predicting ride-hailing fares.

## Figures and Tables

**Fig. 11:**
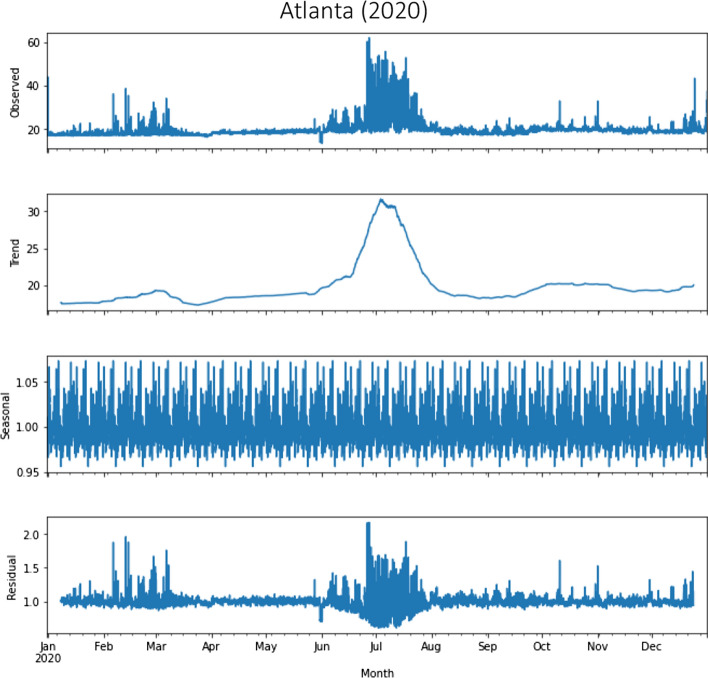
Seasonal decomposition using moving averages in Atlanta in the year 2020

**Fig. 12:**
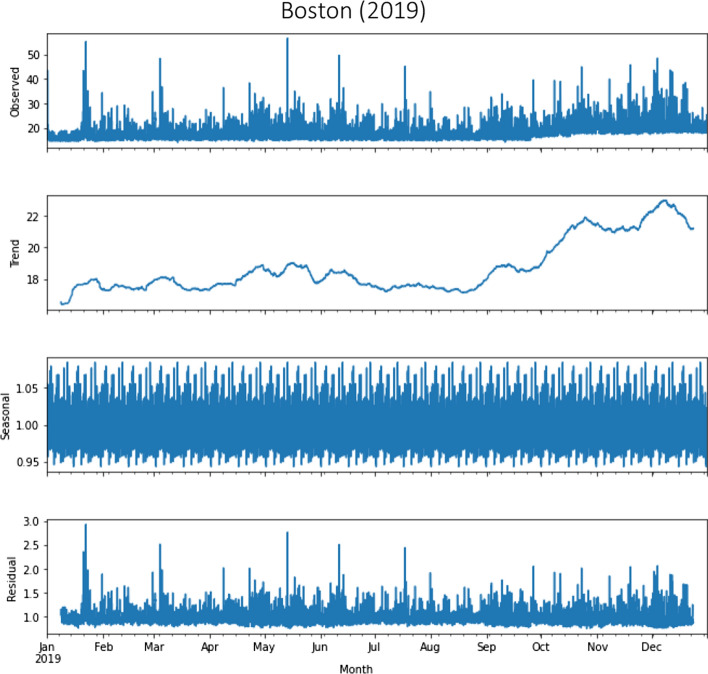
Seasonal decomposition using moving averages in Boston in the year 2019

**Fig. 13:**
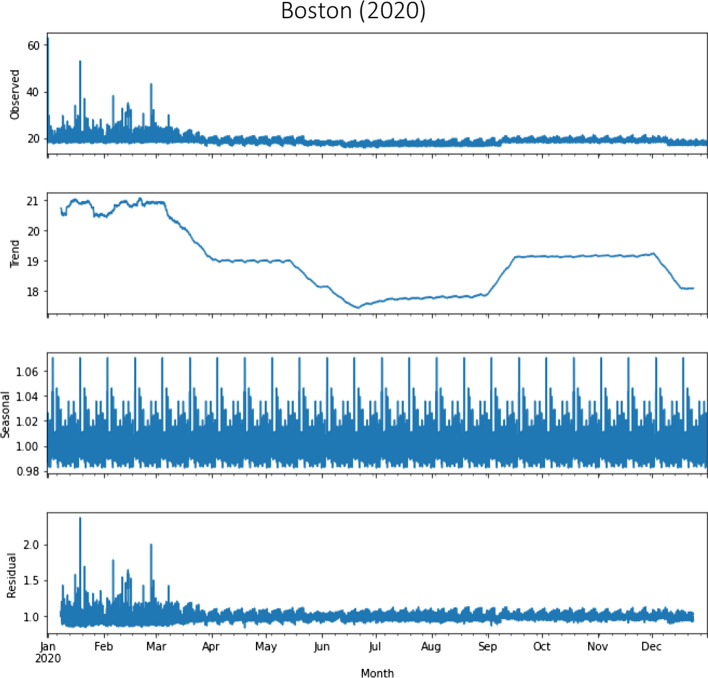
Seasonal decomposition using moving averages in Boston in the year 2020

## Appendix

- Time Series Decomposition with Moving Averages. (See Figs 10, 11, 12 and 13)
